# Feasibility of a stepped wedge cluster RCT and concurrent observational sub-study to evaluate the effects of modified ward night lighting on inpatient fall rates and sleep quality: a protocol for a pilot trial

**DOI:** 10.1186/s40814-015-0043-x

**Published:** 2016-01-07

**Authors:** Satyan R. Chari, Simon Smith, Alison Mudge, Alex A Black, Mariana Figueiro, Muhtashimuddin Ahmed, Mark Devitt, Terry P. Haines

**Affiliations:** 1 Physiotherapy Department, Monash University, Melbourne, Victoria Australia; 2 Safety and Quality Unit, Metro North Hospital and Health Service (MNHHS), Royal Brisbane and Women’s Hospital, Brisbane, Queensland Australia; 3 Centre for Accident Research and Road Safety—Queensland (CARRS-Q), Queensland University of Technology (QUT), Brisbane, Queensland Australia; 4 Faculty of Health, School of Psychology and Counselling, QUT, Brisbane, Queensland Australia; 5 Internal Medicine and Aged Care, MNHHS, Royal Brisbane and Women’s Hospital, Brisbane, Queensland Australia; 6 School of Optometry and Vision Science, Institute of Health & Biomedical Innovation, Queensland University of Technology, Brisbane, Australia; 7 Lighting Research Centre, Rennsselaer Polytechnic Institute, Troy, New York USA; 8 Architectural and Engineering Services, Metro North Hospital and Health Service (MNHHS), Royal Brisbane and Women’s Hospital, Brisbane, Queensland Australia; 9 Allied Health Research Unit, Monash Health, Melbourne, Victoria Australia

**Keywords:** Falls, Hospital, Feasibility, Randomised trial, Environmental modification, Lighting

## Abstract

**Background:**

Falls among hospitalised patients impose a considerable burden on health systems globally and prevention is a priority. Some patient-level interventions have been effective in reducing falls, but others have not. An alternative and promising approach to reducing inpatient falls is through the modification of the hospital physical environment and the night lighting of hospital wards is a leading candidate for investigation. In this pilot trial, we will determine the feasibility of conducting a main trial to evaluate the effects of modified night lighting on inpatient ward level fall rates. We will test also the feasibility of collecting novel forms of patient level data through a concurrent observational sub-study.

**Methods/design:**

A stepped wedge, cluster randomised controlled trial will be conducted in six inpatient wards over 14 months in a metropolitan teaching hospital in Brisbane (Australia). The intervention will consist of supplementary night lighting installed across all patient rooms within study wards. The planned placement of luminaires, configurations and spectral characteristics are based on prior published research and pre-trial testing and modification. We will collect data on rates of falls on study wards (falls per 1000 patient days), the proportion of patients who fall once or more, and average length of stay. We will recruit two patients per ward per month to a concurrent observational sub-study aimed at understanding potential impacts on a range of patient sleep and mobility behaviour. The effect on the environment will be monitored with sensors to detect variation in light levels and night-time room activity. We will also collect data on possible patient-level confounders including demographics, pre-admission sleep quality, reported vision, hearing impairment and functional status.

**Discussion:**

This pragmatic pilot trial will assess the feasibility of conducting a main trial to investigate the effects of modified night lighting on inpatient fall rates using several new methods previously untested in the context of environmental modifications and patient safety. Pilot data collected through both parts of the trial will be utilised to inform sample size calculations, trial design and final data collection methods for a subsequent main trial.

**Trial registration:**

Australian New Zealand Clinical Trials Register (ANZCTR): ACTRN12614000615684 (cluster RCT) and ACTRN12614000616673 (observational sub-study).

Date Registered: 10 June 2014 (both studies).

Protocol version: 1.2 (Dated: 01 June 2014)

Anticipated completion: September 2015

Role of Trial Sponsor: The named sponsor for this investigator-initiated trial was the Director of the Royal Brisbane and Women’s Hospital (RBWH) Safety and Quality Unit (Therese Lee, Phone: +61 7 3646 8111). The principal investigators, SC and MA, are employed by the RBWH Safety and Quality Unit. The trial sponsor has no involvement in any aspects of study design, conduct or decision to submit the report for publication. AM and MD are employed by other departments in the same organisation.

## Background

Falls are among the most frequently reported adverse events among hospital patients globally [1, 2], with reported rates summarised as ranging from 1 to 9 falls per 1000 bed days [3]. Importantly, approximately 30 % of all falls will result in some level of physical harm to patients [4]. While falls with serious injuries are less frequent [5], these incidents are associated with substantial health system costs [6, 7] and considerable elevation in morbidity and mortality [8, 9]. Falls without injury are also of concern as they can instil a fear of falling among older people [10], which in turn may contribute to future activity limitation and decline in function [11]. Overall, patients who fall in hospital are more likely to experience longer hospital stays [12], poorer outcomes at discharge [13] and require costly care [14]. Hence, prevention of these incidents is an important priority for health facilities.

A range of patient-related or intrinsic factors are known to influence the risk of falls in hospital including advanced age, illness status, functional limitations, cognitive impairments and visual deficits [3, 15, 16]. In addition to these, individual fall events may be also be precipitated by extrinsic variables, such as the presence of slip and trip hazards [5]. Intervention trials have attempted to reduce falls by addressing various modifiable risk factors [4, 17–25], and some have demonstrated effectiveness [4, 17–20]. Yet the inconclusive and negative results yielded by other studies [21–25] highlight the challenges inherent in addressing the problem of falls in the hospital setting. Hospital admissions are typically short; at-risk patients are often acutely ill and present with multiple co-morbidities [26]. As a result, the frequency and intensity of interventions necessary to reverse many patient-level risk factors may be higher than what is feasible in busy and challenging clinical environments. In this regard, targeting the environmental contributors for falls [27–29], specifically through the implementation of environmental redesign measures, could offer some benefits over interventions delivered directly to patients.

In comparison to patient-level intervention strategies, measures involving environmental redesign would be associated with higher initial (fixed) costs but lower ongoing (variable) costs. The initial outlay for environmental modifications may be further lowered if incorporated in routine refurbishments or in the construction of new facilities [30]. More significantly, issues that hamper the delivery of most patient-level interventions, such as fluctuating workloads and competing clinical priorities, would not diminish the effectiveness of environment redesign strategies. Nevertheless, there has been scant research on environmental redesign in the context of fall prevention in the hospital setting and no experimental trials except for one pilot study which evaluated the effects of an environmental redesign intervention (modified flooring) on injuries from falls in hospital wards [31].

### Environmental lighting and the risk of falls among admitted patients

Environmental lighting at night has long been suspected to exert an important influence on the risk of falls among older hospital patients [32] and the prevalence of visual impairment in people aged over 60 [33] is one of the central issues. Age-related deterioration in the visual system is most usually manifested as a loss of visual acuity; however, problems such as reduction in the visual field, lowered sensitivity to luminance contrast, and increased sensitivity to glare are not uncommon [34]. Ageing is further associated with decreasing efficiency in the visual system’s capacity to shift from photopic (normal human vision in brightly lit conditions) to mesopic and scotopic states (vision in transitional and dark conditions, respectively) [35]. While loss of acuity is often correctable, narrowed visual field, degraded contrast sensitivity, increased sensitivity to glare and slower adaptation responses to illumination changes are more difficult to treat. These problems would present challenges for older people as they may be more inclined to ambulate at night [36, 37] and could explain the considerable number of night time inpatient falls that are reported [38].

Recognising the widespread nature of age-related deterioration in visual function, some authors have called for better lighting to reduce falls in older populations [39] and this would equally apply to the design of hospital environments. However, bright lighting in patient rooms at night is generally discouraged by applicable design standards [40]. Yet, patients who ambulate at night may require higher light levels than what is normally provided in sleep environments in order to safely ambulate [41]. In shared patient rooms where both sleep and ambulatory activities may overlap, the disruptive effects of excessive night lighting [42, 43] cannot be ignored. Hence, a more considered approach is necessary when designing night lighting profiles for older hospitalised patients [44].

### Proposed night lighting intervention to reduce falls among admitted patients

A series of completed investigations have examined the effects of various forms of indoor night lighting for older people [45–47]. These studies found that at comparable low illumination levels, indoor lighting systems that enhanced horizontal and vertical (H/V) spatial elements were associated with the highest levels of performance on standardised tests of gait and balance. H/V cues in the above trials were generated through the use of light emitting diode (LED) strips installed in linear arrays around room design features of known dimensions and orientation, specifically the bathroom doorframe. A follow-up field study [48] assessed the suitability of a similar LED-based night lighting scheme to address the issue of insufficient lighting in residential care settings [49] and to assist in fall prevention efforts. The temporary installations consisted of the LED-based H/V lighting as per prior lab trials, but with the inclusion of additional elements to meet the functional lighting needs of older people within the room and bathroom environment [50]. These additional components were targeted illumination (a) within the attached bathroom (in sections above the toilet and sink) and (b) under the resident’s bed to assist residents in finding footwear and to identify hazards in the immediate vicinity of the bed. Illumination levels in various locations within the room were also objectively measured and reported on [51]. The study reported that residents and nursing staff responded positively in post-trial interviews with questions spanning acceptability of the solution and participants’ subjective perception of lighting improvement. Nursing staff also indicated that they would be less likely to switch on overhead lights to conduct patient observations due to the presence of the night lighting. These findings have been integrated into evidence-based recommendations for 24-h variable lighting systems in residential care settings with specific reference to the needs of older people with dementia [52]. The LED luminaires used in this series of research were reported as being of ‘amber’ colour and specifically selected to align with extensive literature on the photobiologic effects of light [43, 53–55] and the perceptual-, health- and mood-related benefits [56, 57] associated with the use of long wavelength light. Notably, the measured illuminance readings from the field trial [48, 51, 58] are within the recommended ranges for patient room night lighting as per Australian and New Zealand Interior lighting standards for hospitals [40].

**Fig. 1:**
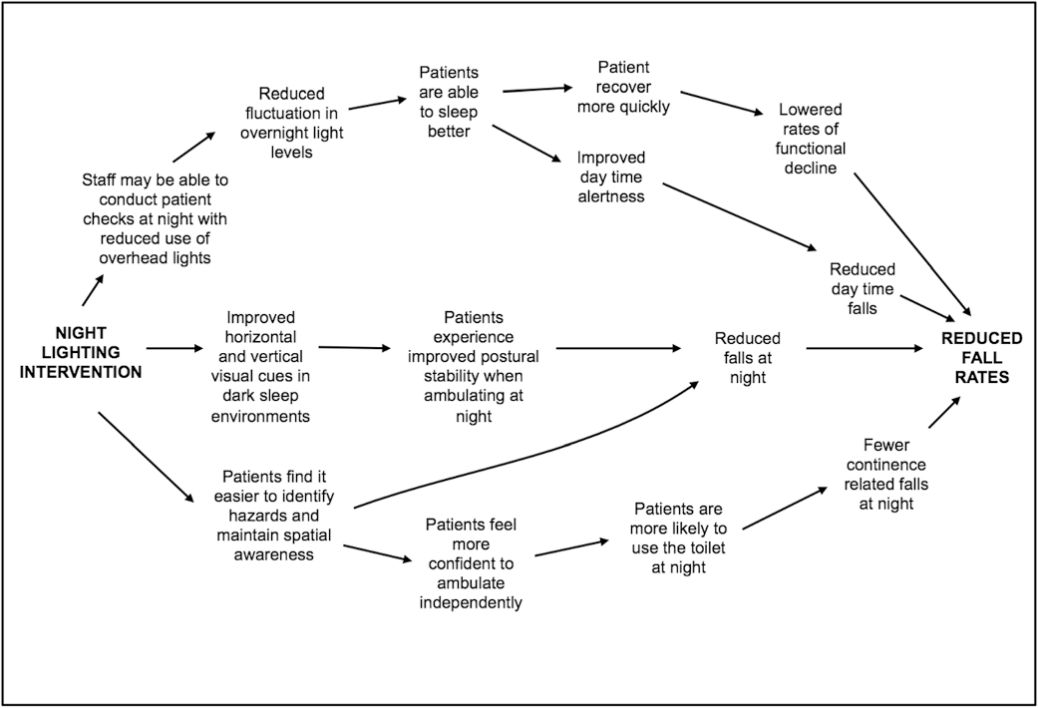
Postulated mechanisms of effect via which the night lighting intervention could reduce rates of falls on hospital wards

We hypothesised that the introduction of a LED-based night lighting scheme (the ‘intervention’) in hospital rooms may contribute to fewer falls through several mechanisms (Fig. 1), but primarily through safer overnight ambulation among patients and reduced sleep disruption. We also hypothesised that the intervention would reduce peak lighting levels at night without impeding overnight usage of toilets by patients and would enhance the perceived environment from a patient perspective.

### Objectives

The primary objective of this pragmatic pilot trial is to understand the feasibility of the stepped wedged cluster randomised controlled trial design to test the effects of introducing modified night lighting across inpatient wards to reduce fall rates. A secondary objective is to study the feasibility of and value gained from a range of proposed data collection methods aimed at understanding the effects of the intervention at the patient level. The pilot data will inform sample size calculations, assist in the confirmation of intervention features, guide the selection of data collection methods and finalise the cost-effectiveness modelling approach to be adopted for a subsequent main trail.

## Methods/design

### Setting

This pilot trial will be conducted in inpatient wards at the Royal Brisbane and Women’s Hospital (RBWH). The RBWH is a 929-bedded publically funded quaternary and tertiary referral teaching hospital in a metropolitan region in southeast Queensland, Australia and is part of the Metro North Hospital and Health Service. A centrally coordinated fall prevention program has been in place at the RBWH for several years, and a consistent approach to fall risk assessment and management is followed across all inpatient wards.

### Design

We will pilot a single-centre-stepped wedge cluster randomised controlled trial (RCT) and a concurrent observational sub-study. The pilot RCT will be aimed at evaluating the effects of the intervention on ward-level fall rates and average length of ward admissions compared to usual care. The mode, quality and quantity of care delivered to patients in intervention wards will not be altered by the pilot trial. The observational sub-study will be conducted with a sample of patients admitted to study wards and will help modelling effects of the intervention on secondary patient- and room-level (environmental) outcomes. The RBWH ethics review board approved this study.

### Design of stepped wedge cluster RCT

**Fig. 2:**
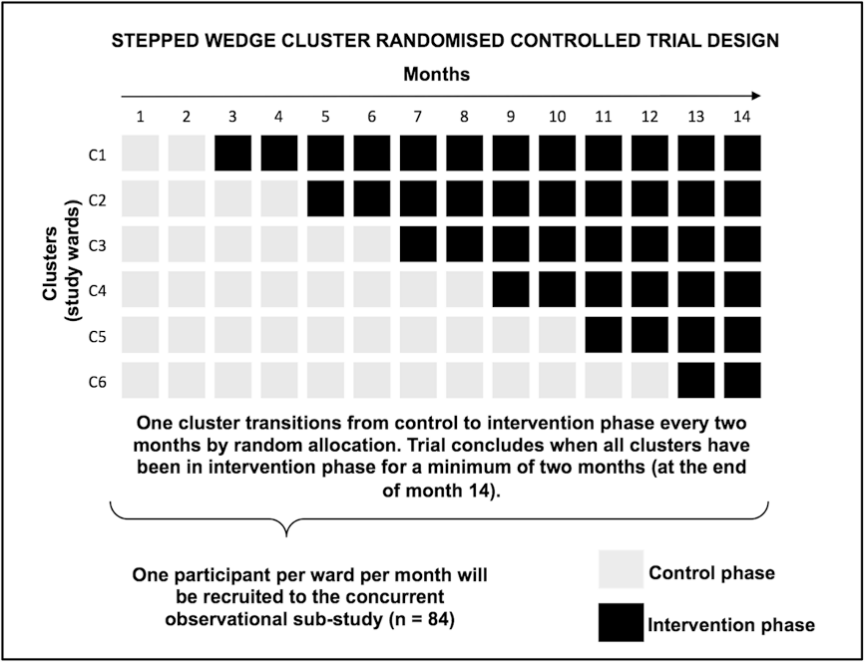
Schematic diagram of study planned progression and trial design

The pilot trial will be undertaken across six RBWH wards (or clusters) and will be conducted over a 14-month period. The pilot cluster RCT will be of a ‘stepped wedge’ design [59], which differs in some important respects from the design of a conventional cluster RCT. A stepped wedge cluster RCT has clusters that provide both control and intervention data. This is achieved by implementing the intervention across all clusters recruited to the study. However, the sequence in which wards cross over from one condition to the other is randomly determined. In this study, all of our study wards will commence as controls at baseline. Figure 2 outlines the pattern in which study wards will transition from control to intervention phases. The first ward to transition to intervention phase will do so 2 months after study commencement and subsequent wards will transition to intervention phase at set intervals (or steps) of 2 months until all wards have transitioned from control to intervention phase. A further period of 2 months of data collection will be undertaken before the study is complete. Thus, all wards will provide a minimum of 2 months of control and intervention data. The stepped wedge cluster RCT design described here is increasingly seen as a suitable design for the pragmatic evaluations of patient safety [60, 61] and service delivery [62] interventions.

### Wards and participants

We will recruit inpatient wards that are located at the RBWH campus (excluding an off-campus rehabilitation ward) with the highest reported rates of patient falls over the preceding 2 years. No specific limits will be applied to recruitment on the basis of service profile, patient demographics or diagnostic grouping. However, plans for ward closure or major physical refurbishments during the study period will be criteria for non-inclusion due to the potential for confounding effects on the pilot trial. Once study wards have been identified, an independent statistician will generate an implementation sequence using computer randomisation software. The sequence of transition from control to intervention phase will not be revealed en bloc and but rather one ward at a time and 2 months prior to the date of transition. This would mean that the first ward to transition from a control to an intervention phase will be revealed to the study team at baseline (month ‘zero’), with the transition to occur at the start of month ‘two’. Similarly, the second ward to cross over will be revealed at the start of month ‘two’ and cross over at the start of month ‘four’. This advance notification of wards due for transitioning to the intervention phase is necessary to allow adequate time for the hospital building and engineering services to complete and test the installations across all rooms in the ward (shared and single) in time for commencement of the intervention phase. Analysis of fall rates will be at the level of the ward and rely on data aggregated from patient incident reports completed by staff and from data coded routinely from the medical record for all admitted patients. A waiver from seeking individual patient-level consent for this component was sought and granted by the RBWH institutional human research and ethics committee.

### Design of concurrent prospective observational sub-study

Detailed patient-level outcomes will be measured by systematic sampling across participating wards throughout the pilot trial as part of a prospective observational sub-study. One patient per ward per month will be recruited from all six included wards (*n* = 84) and we will collect data through a combination of self-report and continuous monitoring, the details of which are described under the ‘outcome measures’ section of this protocol. Written consent will be secured prior to participant recruitment. Patient eligibility will be established by the study research officer (RO) in consultation with the ward nurse manager or shift coordinator at each recruitment point. The RO will be responsible for securing participant consent. Some inclusion/exclusion criteria will apply to recruitment. We will exclude patients who have been admitted for longer than 30 days or are due to be discharged within the following 3 days. Patients that have been recruited to the current study in a previous month will also be ineligible for recruitment. Patients with known cognitive impairment will be excluded, as an important element of this sub-study is to capture data on participant subjective experiences and this would be infeasible among cognitively impaired patients. Due to lack of multi-language versions of the scales used in this study, we will also exclude patients who are not fluent in English. We will exclude patients who are unstable or deemed too unwell to participate. Finally, patients aged less than 65 years on the day of recruitment will also be considered ineligible for recruitment.

Prior to the start of the study, the principal investigator (PI) will produce 84 sets of computer-generated random integer sequences ranging from ‘1’ to the maximum number of bed locations in any ward, for example, ‘35’. These will then be printed and placed in sealed opaque unmarked envelopes and handed over to a RBWH Safety and Quality Unit (SQU) staff member who is not involved with the study. At each recruitment point, the RO will identify, in consultation with ward nursing managers, patients who are eligible to be approached for the study and a list of eligible bed numbers will be compiled and communicated to the nominated SQU staff member. The staff member will then open one envelope and will generate the order of recruitment as per the number sequence in the envelope. This list will be provided to the RO for commencement of recruitment. The PI will monitor recruitment to ensure adherence to the recruitment protocol. The RO will approach the first bed number on the recruitment order; if a patient does not consent to participate in the study, this process will continue until a patient is successfully recruited. This process will be replicated for each ward at every recruitment point.

### Sample size

For the cluster RCT, we are constrained by the available funding as to the number of wards that can be included in the pilot trial (six) and period for which data can be collected (14 months), and hence, the overall anticipated sample is estimated to be approximately 7500 patients. While it is more usual for pilot studies to be conducted with smaller sample sizes, physical design change of this nature has never been undertaken within a stepped wedge cluster RCT. Due to the potential of a variety of operational and logistical challenges in installing new equipment over entire wards, we anticipate that the feasibility of such an approach will only be confirmed by undertaking a rolling sequence of ward installations. In this regard, installations in six wards would provide sufficient opportunities to elicit the full range of practical challenges that may be experienced and afford adequate time to test and modify our implementation approach. Hence, the larger sample size of the study can be considered a secondary feature of this pilot study rather than a primary goal. Within these sample estimates, it is possible to directly calculate the likely effect size that we can detect with 80 % power. Power for parallel cluster randomised trial designs can be calculated by using a conventional power analysis approach and then applying a multiplier to compensate for a design effect, taking the formula [1 + (*n* − 1)*p], where ‘*n*’ = the number of subjects per cluster and ‘*p*’ = the intra-cluster correlation coefficient to take into account the dependency of observations within clusters [63]. However, the stepped wedge design additionally has a ‘within-cluster’ element (that is, all clusters provide intervention and control data), which provides power advantages to a parallel cluster RCT in a similar way to the comparison of paired with unpaired *t* tests. Hence, a power calculation specific to stepped wedge designs is required. One previously described approach highlights that stepped wedge designs could reduce the required sample size in cluster randomized trials [64], but is based upon the cohort style of stepped wedge design where individual participants are repeatedly measured across the length of the study. This is not consistent with our planned study, which reflects more a cross-sectional style of stepped wedge where individual participants are likely to only have one measurement for their involvement in the study. Fortunately, another power analysis approach based on the cross-sectional style of stepped wedge trial better suited for examining a dichotomous outcome (that is, for comparing patients who fall with those who do not) has been developed [65]. Using this approach, our study has 83 % power to detect an absolute reduction in the proportion of patients who are fallers (patients who experience one or more falls) from 5 % (in control) to 4 % (in intervention) assuming 1071 patients per time period (total *n* ≈ 7500), a coefficient of variation of 0.4, and using six clusters (wards), seven time periods (six steps plus baseline) and a two-tailed alpha of 0.05. This represents a 20 % relative reduction in the proportion of patients who become fallers. We anticipate that the minimum important difference for our intervention is likely to be lower than this since an effect size as low as 5 % relative reduction could still represent a cost-effective solution. Nonetheless, in line with the pilot aims of this trial (primarily focused on feasibility), statistical power and cost-effectiveness are not primary considerations and will be the focus of a subsequent main study if the main aims of this pilot trial are satisfactorily met.

### Intervention

The lighting system specifications and installation scheme to be tested in the present pilot trial are aligned with prior research, described in detail by Figueiro and colleagues [46, 51, 58]. However, extensive in-hospital modelling and testing was undertaken by the investigative team in preparation for the present pilot trial guided by the United Kingdom Medical Research Council guidance on the development and evaluation of complex interventions [66]; certain design modifications were found to be necessary. These modifications were (a) incorporation of aluminium channels and diffusers (top plate) to house the LED luminaires in order to meet in-hospital durability and disinfection requirements, (b) elimination of the motion sensing feature described as part of an earlier trial in residential care settings [48], so as to avoid repeated and potentially disruptive cycles of activation and deactivation in multi-occupancy patient environments and (c) exclusion of under-bed lighting due to the risk of loss of lighting units when hospital beds are relocated, warranty concerns associated with affixing electrical devices to hospital equipment, and the possible confounding effects from the presence of integrated under-bed lighting in certain newer bed models procured at the RBWH. Proposed configurations and placement of remaining lighting elements for the present pilot trial will remain unchanged, although minor variations in installations are anticipated as electricians will need to accommodate for differences in design features and to negotiate any unexpected engineering challenges in individual rooms.

**Fig. 3:**
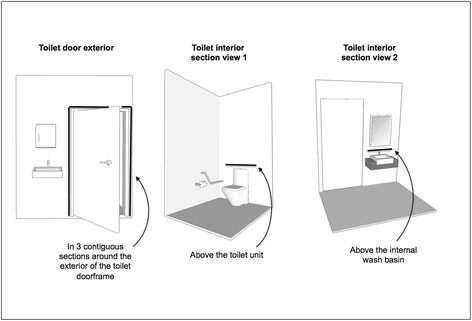
Illustration of in-room locations of lighting intervention and configuration of luminaire installation

We will use commercially available LED strip lighting with an output wavelength of 670 nm (orange colour), which will be housed in aluminium tracks with diffuser cover strips. The lights will be installed in the following three locations across all patient rooms prior to each ward’s transition to the intervention phase:

1. Around the exterior door frame of the attached toilet: To provide a visual reference point for patients attempting to mobilise to the toilet at night, we will install a continuous length of strip lighting around the exterior perimeter of the toilet door frame.
2. Above the washbasin in attached toilets: In a horizontal section below the mirror and above the washbasin.
3. Behind the toilet/adjacent to the toilet: To facilitate easy visualisation of the immediate area around the toilet, we will install one section of lighting behind to the toilet (above the cistern) and in a second section over the grab rail (where a wall-mounted grab rail is installed along the closest adjacent wall). Due to known inter-ward variability in location of toilets, type and location of grab rails within attached bathrooms, it might not be feasible to install grab rail lighting in all locations (such as when the toilet is located equidistant from adjacent walls or if bilateral fold-back grab rails are installed on the posterior wall on either side of the toilet). In such instances, we will make pragmatic decisions on implementation feasibility and variation, which will be comprehensively catalogued by the PI in a study journal. Installation locations are also illustrated in Fig. 3 and should be considered in conjunction with images presented in prior studies [47, 48, 50–52, 58] for approximations of spectral and illumination characteristics.

RBWH electricians will install the LED lighting units and installations will proceed in fully operational ward environments, without bed closures or patient relocations. The PI will coordinate installations with ward managers to ensure that minimal disruptions to normal work flow occur while ensuring that electricians have adequate access to the ward environment to install and test the lighting and control systems. Output levels of individual luminaires have not been specified in the registered protocol as data of this nature was not available prior to commencement. While it is planned that luminaire output will closely follow output levels specified in prior research [48, 51], further calibration will occur during the installation process in consultation with clinical staff and thereafter left unchanged for the duration of the pilot trial. Resultant luminance (emitted light) will be measured in situ after installations are complete and reported on alongside pilot trial results to assist with replication. Activation of the lighting units will be automated to ensure that they are operational overnight (planned operation from 5 PM through to 7 AM). This will help maximise service life and reduce power consumption. The activation and deactivation times have been selected to ensure availability of the lighting during dark periods and so that local seasonal variations of sunrise and sunset times are accounted for. Importantly, none of the existing lighting will be altered in any way, and staff and patients will have full use of existing lighting in patient rooms and toilets if the installed night lighting was considered inadequate or required supplementation; for example, if additional light is required for patients with severe visual impairment, or if a medical emergency occurs.

### Outcome measures

In this pilot cluster RCT, we will test the directionality of intervention effects on ward level ‘rate of falls’ (number of falls per 1000 admitted patient days) and the proportion of patients who have one or more falls as an inpatient. Fall outcome data will be compiled from multiple sources, namely, (a) incident reports completed on the Queensland Health (QH) clinical incident reporting system, (b) periodic contact with the ward nurse unit managers to identify any potentially unreported falls and (c) data routinely extracted by clinical coders from patient medical records after discharge. The PI will extract aggregated clinical incident reports on a monthly basis to update the project database, adding any unreported falls identified by the RO through ward contact. Additional falls coded from medical records, length of stay, ward bed occupancy and general demographic data for patients in study wards will be extracted from the clinical costing and case-mix system maintained by RBWH Health Information Services (HIS). These coded data will be extracted 3 months after the conclusion of the trial. To explore the effect of the intervention specifically on night-time falls, we will also include a secondary sub-analysis of the ‘rate of falls at night’ (falls occurring between the hours of 7 PM and 6 AM) using the same statistical approach described here. We note that the decision to include this secondary analysis is a protocol modification that occurred after trial registration but before data was ready for statistical analysis.

The observational sub-study will help us in understanding the manner in which the modified environment might influence patient level outcomes. Outcome measures will primarily aim to capture data on sleep quality, overnight activity levels, insomnia and daytime sleepiness. Table 1 describes the tools that will be utilised to collect this data.

**Table 1:** Primary outcome measures for observational sub-study

Construct	Tool	Description	Frequency of data collection
Sleep quality and overnight activity levels	Philips Actiwatch 2—Wrist Actigraph	In order to collect objective data on participant sleep quality, sleep fragmentation, total sleep and overall activity levels, we will use wrist actigraphy. Actigraphs are wearable sensors that allow logging of movement data and have been extensive used in clinical research. Actigraphy data has been validated against gold-standard polysomnography methods and offers a reliable tool for measuring sleep outside of a sleep laboratory environment [71].	Days 0, 3, 7 and 12, consisting of one initial interview and a maximum of three follow-up interviews, unless patient is discharged or moved to another room prior to day 12. Days 3, 7 and 12 data collection will occur ±1 day to accommodate for weekends and public holidays.
		We will be using a Philips Actiwatch 2, which is a small, rugged, waterproof wrist worn data logger with long battery life and will provide us with a measure of rest-activity patterns and sleep. The Philips Actiwatch range has been applied in over 30 clinical trials to date including the study of sleep-wake patterns in older acute patients [72, 73].	
		Upon recruitment, the research officer will apply the Actiwatch on the participant’s non-dominant wrist and re-check application and wearing behaviour at every researcher-participant contact point thereafter.	
Daytime sleepiness	Karolinska Sleepiness Scale (KSS) [74]	The KSS is a short 9-item self-report questionnaire that is a measure of a situational sleepiness. The KSS is sensitive to daily changes in levels of sleepiness [75].	As above.
Insomnia	Insomnia Severity Index (ISI) [76]	Insomnia is an important manifestation of sleep disturbance and thus an important construct to measure. The ISI is a brief validated 7-item self-report measure of the individual’s subjective perception of insomnia (sleep onset, maintenance and early and unintended waking) as well as amount of concern generated due to those symptoms. The ISI has been utilised in prior studies on insomnia prevalence in older admitted populations [77].	As above.

A secondary focus will be participant self-reported premorbid sleep quality, vision, hearing, functional status, self-reported causes for disruption to sleep in hospital, participant light dosage levels and overall satisfaction with the room physical environment. Data on environmental lighting (within participant rooms) will be captured continuously using environmental sensors and data loggers. Secondary measures and environmental monitoring modalities are described in Table 2. All measures will be collected at the initial interview. Wrist actigraph and environmental sensors will also be applied at the time of the initial interview. Repeat measures (with the exception of premorbid sleep, current vision and hearing status) will be collected every 3 days (plus or minus 1 day) up to day 12 or discharge/transfer, whichever occurs earlier. The collection of these data will provide a detailed context for understanding potential mechanisms of effect for the intervention.

**Table 2:** Pre-admission participant characteristics and secondary measures for observational sub-study

Construct	Tool	Description	Frequency of data collection
Pre-admission sleep	Epworth Sleepiness Scale	To explore whether any reported insomnia or sleep fragmentation is new or pre-existing, we will administer the Epworth Sleepiness Scale (ESS). The ESS is a widely used and valid 8-item self-administered instrument for measuring for excessive daytime sleepiness [78]. Excessive daytime sleepiness is associated with a range of disorders and has been associated with falls in certain older groups [79].	Initial interview only (day 0)
Vision impairment	Impact of Visual Impairment Scale	As participant visual status would influence the benefit derived from an environmental lighting solution we will ask participants to self-rate the functional impact of any visual impairment using the Impact of Visual Impairment Scale (IVIS). The IVIS is a widely cited and validated brief five-item instrument [80], which measures impact of vision impairment in terms of difficulties with simple tasks. Individual IVIS items are suited to older people in the inpatient setting and measure constructs relevant to study aims.	As above.
Hearing impairment	Hearing Handicap Inventory for the Elderly—Screening	The presence of hearing impairment is of secondary interest to contextualise data on causes of sleep disruption as patients with hearing impairment may be less affected by environmental noise than those patients with unimpaired hearing. We will measure the functional impact of hearing impairment among study participants by using the Hearing Handicap Inventory for the Elderly—Screening (HHIE-S). The HHIE-S is a short ten-item measure of the social, emotional and functional impacts of hearing impairment rather than a definitive measure of the degree of hearing impairment [81]. However, as a self-report measure, it has demonstrated excellent reliability and specificity in detecting the level of impact of hearing loss [82].	As above.
Self-reported causes for disruptions to sleep	Interview questions formulated by investigative team.	Sleep disruptions can occur due to multiple factors in addition to light levels. Therefore, we will ask patients to what degree their sleep was disrupted by specific causes (rated on a 7-point Likert-type scale ranging from ‘Never’ to ‘Constantly’).	Initial interview (day 0) and repeated on days 3, 7, and 12 Maximum of three follow-up interviews, unless patient is discharged or moved to another room prior to day 12. Follow-up data collection will occur ±1 day to accommodate for weekends and public holidays.
		The specific items are ‘Pain or Discomfort’, ‘Anxiety and Thoughts’, ‘Feeling unwell’, ‘People talking in your room’, ‘Alarms and sounds from medical devices’, ‘Sounds made by other patients’, ‘Bright lights being left on overnight’, ‘Bright lights being switch on while you sleep’, ‘Staff providing care to you’, ‘Staff providing care to others’ and ‘Volume of someone else’s television’.	
		The current 11 items represent a refinement over a previous version that was developed after a review of the hospital sleep literature and modified following with admitted patients in the prior (unpublished) modelling research conducted by Chari S et al. to inform the present pilot RCT.	
Functional status	5-item Barthel Index	We will measure patient functional status using the 5-item Barthel Index [83] to capture any variations in functional status as this would influence the interpretation of mobility data collected through continuous direct monitoring. The Barthel Index is a widely used, valid and accepted tool for screening and assessing independence in activities of daily living (ADL) in geriatric settings, including older hospitalised patients.	As above.
Satisfaction with the room environment	Multiple choice question and free-text	To evaluate overall participant satisfaction with the physical environment of the room and bathroom, we will ask participants to rate their level of satisfaction on a 5-point Likert-type scale ranging from ‘Very satisfied’ to ‘Very Dissatisfied’. We will ask participants to suggest potential improvements to the physical environment that could help patients to feel more confident to move about safely in their room and bathroom, to sleep better or to assist patients in any other way.	As above.
Participant light dosage	Philips Actiwatch 2—integrated light sensor	The inbuilt light sensor will enable measurement of dosage of ambient white light over a 24-h period. We will set the sampling rate to one measurement every 30 s.	Continuous measurement (commenced at initial interview and continued for period of follow-up
Overnight maximum lighting levels and variation in patient room and toilet.	HOBO U12-012 Light Data Logger	As the modified lighting will be installed both inside the patient room and attached toilet, we will monitor variations in overnight lighting levels using a data logger (Onsetcomp HOBO U12-012) mounted on the wall in the patient room and toilet. The HOBO U12-012 data logger is a high-frequency, high-resolution device capable of a measuring range between 1 and 3000 lumens/square foot. As the Actiwatch sensor will be the primary measure of participant white light dosage, we will affix the data logger outside the immediate patient bedside environment (outside the area circumscribed by patient privacy curtains) in order to measure overall variation in room lighting profile. We will set the sampling rate to one measurement every 30 s.	As above.
Frequency with which toilet doors are opened and closed overnight	Onsetcomp HOBO UX90-001—State Change Data logger	In order to understand whether participants may have been exposed to the modified lighting environment within the toilet overnight, we will log the times of door opening and closing. This will be done through an unobtrusive door mounted data logger that measures contact with a magnetic latch (Onsetcomp HOBO UX90-001). Thus, all state change events (door opening and closing) will be captured. The HOBO UX90-001 is a high-capacity data logger appropriate to measuring simple state changes and will allow us to contextualise activity levels at night.	As above.

A final issue relates to the measurement of intervention fidelity. While intervention delivery itself does not require monitoring in this trial due to the automated nature of the intervention, it will be important to understand whether patient and staff usage of existing lighting is different before and after installation. Undertaking night-time visual observations was not considered possible within the limited scope of this pilot trial. We therefore sought to collect data on lighting usage through the implementation of automated environmental monitoring solutions. ‘Illuminance’ data (incident light on a given surface expressed in ‘Lux’ or lumens per square metre) will be collected in both pre-implementation and post-implementation phases through the use of sensors (with data logging capabilities) affixed in rooms and toilets of patients recruited to the observational sub-study. Through comparison of these data with visually validated reference data collected in the study wards, we will be able to classify the majority of room lighting states with a high degree of confidence (that is into ‘non-lit’, ‘LED lighting only’ and ‘operational ceiling lights’ categories). Further, we will be able to identify the timing, frequency and duration of intervals between state changes. While it would be difficult to attribute state changes to patients or staff based upon  this data alone—comparison with patient time-stamped sleep and activity data collected via wrist actigraphs will help us exclude patient action in some instances where the recruited patient was clearly not the agent causing the state change (for example, if actigraphy data indicates the patient was asleep).

### Masking

Research staff responsible for collecting and entering of data for the cluster RCT will not be blinded to the identity of control or intervention wards, and the nature of the intervention precludes blinding among ward staff. For the observational sub-study, selection order will be masked from research staff until a list of eligible patients is compiled for each ward. Patients recruited to the observational sub-study will only be advised that the interview is part of study related to a larger research trial examining links between the hospital physical environment and patient outcomes. However, it is possible that some patients on intervention wards become aware of the intervention during the course of their admission.

### Statistical analysis

The analysis of primary outcomes will be with multi-level mixed effects generalised linear models using a Bernoulli family and logit link for the ‘proportion of patients who become fallers’ outcome and a negative binomial family and log link for the ‘rate of falls’ outcome. In these analyses, patient admissions will be nested within ward in the random effects part of the equation to take account of clustering of data by ward. The fixed part of the equation will examine group effects (intervention vs control phases) and adjust for time period. We will also adjust for seasonal effects on fall rates (that is, we will enter a covariate based on how many falls there were on each ward over the same time period over the previous 2 years). In relation to the observational sub-study, we will employ a full range of descriptive statistics to model the main features and distribution characteristics of the final data set. We will explore differences in patient sleep data and self-reported sleep and mobility behaviour collected over control and intervention periods through both qualitative and quantitative methods. Longitudinal data collected through actigraphy and environmental sensing will be subjected to exploratory analysis using pattern recognition and data filtering algorithms to classify the raw outputs against specific activity and event markers. Aggregated environmental sensing data will be graphically modelled through the development of scatter plots and histograms for visual identification of patterns and differences between control and intervention data. Categorical data on the causes of sleep disruptions collected through the patient interview tool will additionally be subjected to content [67] and thematic [68] analysis. Concurrently, we will undertake multi-level mixed effect generalised linear modelling with the quantitative data from the observational sub-study, using a Gaussian family and identity link for normally distributed continuous data. The random effects would be subjected to nested analysis; however, the fixed effect cannot adjust for seasonal variation, as we will not have access to any prior data of this nature.

### Economic analysis

Upon completion of the pilot trial, we will attempt cost-effectiveness analysis with available data through the use of a decision-analytic model to estimate the costs and health effects that patients would experience with and without the intervention [69]. The expected change in costs and change in effects prompted by this intervention will be used to estimate incremental cost-effectiveness ratio (ICER), which provides a summary measure of ‘value for money’. This allows for a comparison of the night lighting against other health care interventions so long as the measures of health effect are comparable and can assist in the estimation of health interventions that offer the best value for money. The completion of economic modelling from the pilot data will underpin the decision to progress to a main trial and also inform the final economic modelling approach to be adopted.

### Post-implementation staff surveys

In line with pilot aims of this trial, we seek to concurrently understand whether the characteristics of the intervention and chosen implementation methods may be improved from the perspectives of clinicians providing care to patients on intervention wards and of ward managers. This will involve short face-to-face interviews with a sample of ward staff after the pilot trial is complete. The interviews will seek to establish the level of disruptive impact experienced by the ward while installations were undertaken, to gauge overall staff satisfaction with the intervention (after a period of incorporation into normal practice upon conclusion of the pilot trial) and to finally identify ways in which the implementation approach and intervention characteristics may be improved. The scope and focus of post-implementation interviews will be informed by the specific issues and installation challenges noted by the investigations to have arisen over the course of the pilot trial. Accordingly, a separate submission to the responsible institutional review board will be made prior to commencement of post-trial interviews.

### Trial safety and data management

Project investigators will monitor implementation and outcomes for the duration of the pilot trial. The PI will monitor adverse event rates in intervention wards on a continuous basis. All fall-related adverse events resulting in injury will further be analysed individually to evaluate whether the intervention was a contributory factor. All such investigations will be escalated to the Data Safety Management Committee (DSMC) and Human Research Ethics Committee (HREC). The DSMC will be established composed of one experienced clinician with experience in acute care who is not a member of the project investigative team and is not invested in study outcomes (external to the RBWH), an investigator from the research team, an RBWH patient safety representative and a senior clinician representative from the RBWH. This committee will review the interim results of the pilot trial and review any serious adverse events or unexpected outcomes related to the pilot trial. Reporting of any adverse events or unexpected outcomes will be forwarded to the HREC when received. The investigative team member on the committee will be the nominee for ensuring all communications and escalations to the HREC.

In order to avert any unforeseen increase in falls attributable to the intervention, we will monitor for any elevations in falls in study wards after transition to an intervention phase. An elevation of 30 % or more over the previously reported peak in the preceding 12 months will be the threshold for further investigation for potential attribution to the intervention. Findings will be escalated to the project governance group, the chair of the HREC and DSMC nominee for a decision on study continuance or cessation. If a decision for cessation is made for this reason, the lighting will be disabled centrally in all wards followed by physical removal of installations over time.

Data will be collected in an identifiable form and retained as such until linkage of all pilot trial data is completed, upon which the entire dataset will be de-identified prior to analysis. Data storage will be managed in line with Queensland Health data retention and disposal policies and HREC recommendations.

## Discussion

This study will be one of the first pilot investigations into the feasibility of the stepped wedge cluster RCT design to study the effects of hospital environmental modification on inpatient fall rates. The pilot trial also includes a number of novel environmental sensing modalities for data acquisition that are expected to generate new insights into the lighting of hospital wards before and after introduction of the night lighting intervention and potential impacts on night-time patient mobility behaviour and lighting exposure. The completion of this work would provide impetus to further trial-based research aimed at generating high-level evidence to inform future hospital facility design and refurbishment efforts. Finally, the conduct of this pilot trial will help confirm the methodological suitability of the stepped wedge cluster RCT design in the testing of environmental modification interventions and clarify scope for progression towards a main trial.

There are a number of limitations of this pilot study. Primary outcomes in this RCT, that is rate of falls and number of patients who become fallers, will be estimated by reviewing incident reports, via periodic contact with ward manager and through post-trial acquisition of routinely coded data from medical records. However, we recognise that incomplete identification of fall incidents is likely [70]. As indicated previously, and to the best of our knowledge, the stepped wedge cluster RCT design has never been applied to the testing of environmental modification in any setting. Due to the untested nature of this approach, the feasibility of this model and possible challenges in undertaking rolling installations in operational wards remains unknown. Therefore, the primary value of this pilot trial will not be in the results of statistical testing undertaken with pilot data, but rather in confirming whether this is a suitable approach to test the effects of hospital environmental change and in generating sufficient pilot data to test the postulated mechanisms of effect, to confirm the feasibility and utility of proposed data collection methods and to finalise intervention features in advance of a larger follow-up trial.

## Trial status

Recruitment ongoing, anticipated completion in September 2015.

## Abbreviations

AS/NZS: Australian Standard/ New Zealand Standard
DSMC: Data Safety Management Committee
H/V: horizontal and vertical
HREC: Human Research Ethics Committee
LED: light emitting diode
PI: principal investigator
RCT: randomised controlled trial
RO: research officer
SQU: Safety and Quality Unit
